# Mesenteric Cyst in Association with Type-II Jejunoileal Atresia

**DOI:** 10.21699/jns.v5i4.462

**Published:** 2017-01-01

**Authors:** Rajat Piplani, Samir Kant Acharya, Nidhi Sugandhi, Deepak Bagga

**Affiliations:** Department Pediatric Surgery, VMMC And Safdarjang Hospital, New Delhi

**Keywords:** Ileal atresia, Mesenteric cyst, Neonate

## Abstract

A rare case of type-II jejunoileal atresia with mesenteric cyst in a neonate is being reported here with a brief review of literature.

## CASE REPORT

A full term five-day old male neonate born by normal delivery weighing 2.7 kg was referred to our hospital with complaints of bilious vomiting and abdominal distention for three days. Examination of the neonate revealed abdominal distention along with visible bowel loops and bilious nasogastric aspirates. Antenatal ultrasound was normal. Erect radiograph of abdomen revealed distended bowel loops along with few air fluid levels and absent distal gas in pelvis suggestive of jejunoileal atresia. On exploratory laparotomy, type-II jejunoileal atresia at proximal ileum with distal mesenteric cyst measuring 4 x 4 cm causing bowel volvulus around it was noted (Fig.1). The cyst was compressing on the distal ileal segment for about 5 cm and could not be separated from it. Hence, ileal derotation and resection of segment of atretic ileum along with distal mesenteric cyst and end to oblique ileo-ileal anastomosis was done. The histopathology report confirmed it a mesenteric cyst. Postoperative course was uneventful. Follow-up of the child at one year is also satisfactory.

**Figure:**
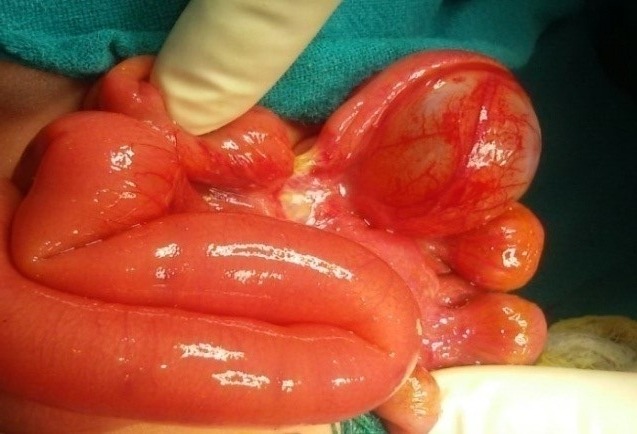
Figure 1: Intra-operative picture showing dilated proximal jejunum with type 2 proximal ileal atresia and distal mesenteric cyst compressing a part of the adjoining ileal segment.

## DISCUSSION

Jejunoileal atresia (JIA) is a major cause of intestinal obstruction in neonates, with an incidence of about 1 in 5000 live births. [1] Jejunal atresia has been associated with number of other congenital malformations such as cystic fibrosis, malrotation, congenital heart disease, Down’s syndrome, congenital dislocation of hips, anorectal and vertebral anomalies, neural tube defects and microcephaly. It can also coexist with other anomalies like biliary atresia, duodenal atresia, colonic atresia, gastric atresia and Hirschsprung’s disease. [2-4] The association of mesenteric cyst with JIA is extremely rare with less than 10 cases reported so far in literature. [5]

Intrauterine mesenteric vascular disruptions to a segment of developed intestine seemed to result in JIA. [6] Intestinal atresias secondary to late intrauterine mesenteric vascular insults are often seen in patients with volvulus, intussusceptions, internal hernia, and tight anterior abdominal wall defects. [3,6] As in our case, mesenteric cyst causing secondary bowel volvulus distally could be the causative factor for the vascular compromise leading to JIA.

We report this extremely rare association of mesenteric cyst causing secondary bowel volvulus and proximal type-II JIA which further reinforces the theory of mechanical obstruction as the cause for JIA.

## Footnotes

**Source of Support:** Nil

**Conflict of Interest:** Nil
